# Clinical profiles of early and tuberculosis-related mortality in South Korea between 2015 and 2017: a cross-sectional study

**DOI:** 10.1186/s12879-019-4365-9

**Published:** 2019-08-22

**Authors:** Jinsoo Min, Ju Sang Kim, Hyung Woo Kim, Ah Young Shin, Hyeon-Kyoung Koo, Sung-Soon Lee, Yang-Ki Kim, Kyeong-Cheol Shin, Jung Hyun Chang, Gayoung Chun, Joosun Lee, Mi Sun Park, Jae Seuk Park

**Affiliations:** 1 Division of Pulmonary and Critical Care Medicine, Department of Internal Medicine, Daejeon St. Mary’s Hospital, College of Medicine, The Catholic University of Korea, Seoul, Republic of Korea; 2 Division of Pulmonary and Critical Care Medicine, Department of Internal Medicine, Incheon St. Mary’s Hospital, College of Medicine, The Catholic University of Korea, Seoul, Republic of Korea; 3 Division of Pulmonary and Critical Care Medicine, Department of Internal Medicine, Ilsan Paik Hospital, Inje University College of Medicine, Goyang, Republic of Korea; 4 Division of Pulmonary and Allergy Medicine, Department of Internal Medicine, Soonchunhyang University College of Medicine, Seoul, Republic of Korea; 5 Division of Pulmonary and Critical Care Medicine, Department of Internal Medicine, Yeungnam University Medical Center, College of Medicine, Yeungnam University, Daegu, Republic of Korea; 6 Division of Pulmonary and Critical Care Medicine, Department of Internal Medicine, College of Medicine, Ewha Womans University, Seoul, Republic of Korea; 7 Division of TB Epidemic Investigation, Korea Centers for Disease Control and Prevention, Osong, Republic of Korea; 8 Division of Pulmonary Medicine, Department of Internal Medicine, Dankook University College of Medicine, 119 Dandae-ro, Dongnam-gu, Cheonan, 31116 Republic of Korea

**Keywords:** Private-public mix, PPM, Death, Elderly, Korea

## Abstract

**Background:**

Although the incidence of tuberculosis (TB) has decreased in South Korea, the mortality rate remains high. TB mortality is a key indicator for TB control interventions. The purpose of this study was to assess early and TB-related mortality during anti-TB treatment and describe the associated clinical characteristics.

**Methods:**

A multicenter cross-sectional study was performed across South Korea. Patients with pulmonary TB who died during anti-TB treatment and whose records were submitted to the national TB surveillance system between 2015 and 2017 were enrolled. All TB deaths were categorized based on cause (TB-related or non-TB-related) and timing (early or late). We identified statistical associations using the frequency table, chi-square test, and binary logistic regression.

**Results:**

Of 5595 notifiable mortality cases, 3735 patients with pulmonary TB were included in the analysis. There were 2541 (68.0%) male patients, and 2935 (78.6%) mortality cases were observed in patients older than 65 years. There were 944 (25.3%) cases of TB-related death and 2545 (68.1%) cases of early death. Of all cases, 187 (5.0%) patients were diagnosed post-mortem and 38 (1.0%) patients died on the first day of treatment. Low body mass index (adjusted odds ratio (aOR) = 1.26; 95% confidence interval (CI) = 1.08–1.48), no reported illness (aOR = 1.36; 95% CI = 1.10–1.68), bilateral disease on chest X-ray (aOR = 1.30; 95% CI = 1.11–1.52), and positive acid-fast bacilli smear result (aOR = 1.30; 95% CI = 1.11–1.52) were significantly associated with early death, as well as TB-related death. Acute respiratory failure was the most common mode of non-TB-related death. Malignancy was associated with both late (aOR = 0.71; 95% CI = 0.59–0.89) and non-TB-related (aOR = 0.35; 95% CI = 0.26–0.46) death.

**Conclusions:**

A high proportion of TB death was observed in elderly patients and attributed to non-TB-related causes. Many TB-related deaths occurred during the intensive phase, particularly within the first month. Further studies identifying risk factors for different causes of TB death at different phases of anti-TB treatment are warranted for early targeted intervention in order to reduce TB mortality.

## Background

An estimated 1.3 million people died due to tuberculosis (TB) in 2017, making TB one of the leading causes of death due to an infectious agent worldwide [1]. The World Health Organization’s (WHO) End TB target is a 95% reduction in the number of deaths due to active TB between 2015 and 2035 [2]. In South Korea, the TB rate has decreased significantly, with a 5.2% annual reduction in the incidence of newly reported TB cases from 2011 to 2016; however, South Korea has the highest TB incidence and mortality rates among the high-income countries [3]. In 2016, the total number of reported cases was 39,245, with an incidence rate of 78.8 persons per 100,000; the mortality rate was 5.1 persons per 100,000 [4]. As South Korea becomes an older-aged society, TB mortality and incidence are rapidly increasing among those over 60 years old; this is a huge obstacle to national TB control [5].

A review [6] of the risk factors associated with death during anti-TB treatment, which include human immunodeficiency virus (HIV) positivity, old age, comorbidities, and use of alcohol and drugs, indicates that there are differences in risk factors among regions with low and high incidence of TB. Due to a low prevalence of HIV infection [7, 8] and an intermediate TB burden, South Korea requires a different strategy to control TB mortality. In addition, the causes of TB mortality may differ depending on the phase of anti-TB treatment; however, there are only few studies investigating early deaths, defined as death occurring within the first 2 months of anti-TB treatment [9–12]. TB mortality is a key indicator for the national TB control program in South Korea. Further studies using nationwide data are required to better understand TB mortality, hence leading to opportunities for public health intervention that can decrease TB mortality and improve treatment outcomes.

With the introduction of the nationwide health insurance system in 1989, TB control in South Korea began to transition from a public health center-based program to a private hospital-based program [13]. In 2011, a public–private mix (PPM) collaboration model was implemented as a national TB control strategy. We collected data of TB mortality cases at PPM-participating hospitals for monitoring and evaluation. The purpose of this study was to assess early and TB-related mortality during anti-TB treatment and describe the associated clinical characteristics.

## Methods

### Study population

We conducted a multicenter cross-sectional study of TB mortality during anti-TB treatment within the PPM collaboration model in South Korea. Patients with pulmonary TB who died during anti-TB treatment and whose data were entered into the Korean National TB Surveillance System (KNTSS) [14] at PPM hospitals across South Korea between 2015 and 2017 were enrolled. The government implemented a PPM collaboration model in 2011 [3]. Through PPM collaboration, comprehensive management of TB patients is provided by TB specialist nurses dispatched to private PPM hospitals; this management includes cases studies, administration of medication during the infectious period, management of side effects until completion of the treatment, and contact examination among family members. More than 210 TB specialist nurses at 127 PPM hospitals and 236 public health officials at 254 public health centers across the country are working under the PPM projects. Sixty-six percent of new TB patients who were notified across the country were treated at PPM hospitals in 2016.

The inclusion criteria were as follows: adult patients over 18 years of age, patients diagnosed with pulmonary TB, patients who died from any cause during anti-TB treatment, and patients who started an initial standard anti-TB regimen. The exclusion criteria were as follows: patients with drug-resistant TB, patients with miliary TB or extrapulmonary TB, patients who did not receive initial standard anti-TB regimen, and patients with anti-TB treatment duration of more than one year.

Patients with drug-sensitive TB underwent a 6-month standard treatment regimen recommended by the Korean Guidelines for TB [15], which consists of a 2-month initial phase of isoniazid, rifampicin, ethambutol and pyrazinamide followed by a 4-month continuous phase of isoniazid, rifampicin, and ethambutol. Alternatively, a 9-month standard regimen with isoniazid, rifampicin, and ethambutol can be administered. Anti-TB drugs were self-administered with the support of TB specialist in the PPM project.

### Data collection

In South Korea, TB notification is mandatory when a physician diagnoses or treats a patient with confirmed or suspected TB. All the TB patients are followed during the anti-TB treatment under the PPM project, and their follow-up is closed once a final treatment outcome, defined by the WHO, has been notified to the KNTSS. After identifying death as a final outcome, TB specialist nurses at each hospital complete a mortality case report form. We retrospectively collected the clinical, radiographic, and microbiological data for each mortality case. Since follow-up after completing treatment was not possible under the PPM project, we could not identify post-treatment mortality cases for TB survivors. We stratified age into 5 groups: ≤ 49, 50–59, 60–69, 70–79, and ≥ 80. Those who have smoked < 100 cigarettes per lifetime were defined as never smokers. Those who did not smoke and drink during the last one year were defined as ex-smokers and non-drinkers, respectively. Men and women who consumed at least five and four drinks, respectively, on a single occasion in the last month or had alcohol use disorder were defined as heavy drinkers.

### Definition of death

The WHO definition of TB death was used in this study, and is defined as patients with TB who died from any cause during anti-TB treatment [16]. Each TB death was categorized by cause (TB-related or non-TB-related death) and timing (early or late death). TB-related death was confirmed by death certificate or medical records from the physician-in-charge. If another cause of death was determined, mortality was classified as non-TB-related. The mode of non-TB-related death was also recorded. In addition, mortalities were divided into groups of early and late death according to whether death occurred within the initial 2-month intensive phase or during the continuous phase of anti-TB treatment, respectively.

The mode of death for non-TB-related death was also collected. Malignant neoplasm included metastatic solid malignancy, leukemia, lymphoma, and chronic refractory hematologic diseases. Acute respiratory failure included acute exacerbation of chronic respiratory diseases. Patients who were diagnosed with pneumonia or aspiration pneumonia and complicated with respiratory failure or septic shock were categorized into pneumonia. Sudden cardiac death included ischemic heart disease, arrhythmia, pulmonary thromboembolism, acute hear failure, and aortic dissection. Senility included elderly patients with malnutrition, dementia, and poor general condition. Unknown category included death at home or another institution and death on arrival at emergency room.

### Statistical analysis

Continuous variables are presented as mean and standard deviation, whereas discrete variables are presented as frequency and percentage. In order to compare the differences between TB-related and non-TB-related death, we performed univariate analysis using the chi-square test and binary logistic regression. We also compared early death with late death accordingly. Subsequently, we selected age, gender and other clinical variables with *P* values < 0.20 based on the univariate analysis and performed multivariate binary logistic regression to evaluate the possible association between variables and predefined TB mortality subset. The calibration of the prediction model was assessed using the Hosmer-Lemeshow goodness-of-fit test (*P* < 0.05 was considered as indicating a statistically significant lack of fit). For regression, unknown data were regarded as missing values. A *P* value of 0.05 was considered statistically significant. All statistical analyses were performed using SPSS software (Statistical Product and Service Solutions, Chicago, IL, USA).

## Results

**Fig. 1:**
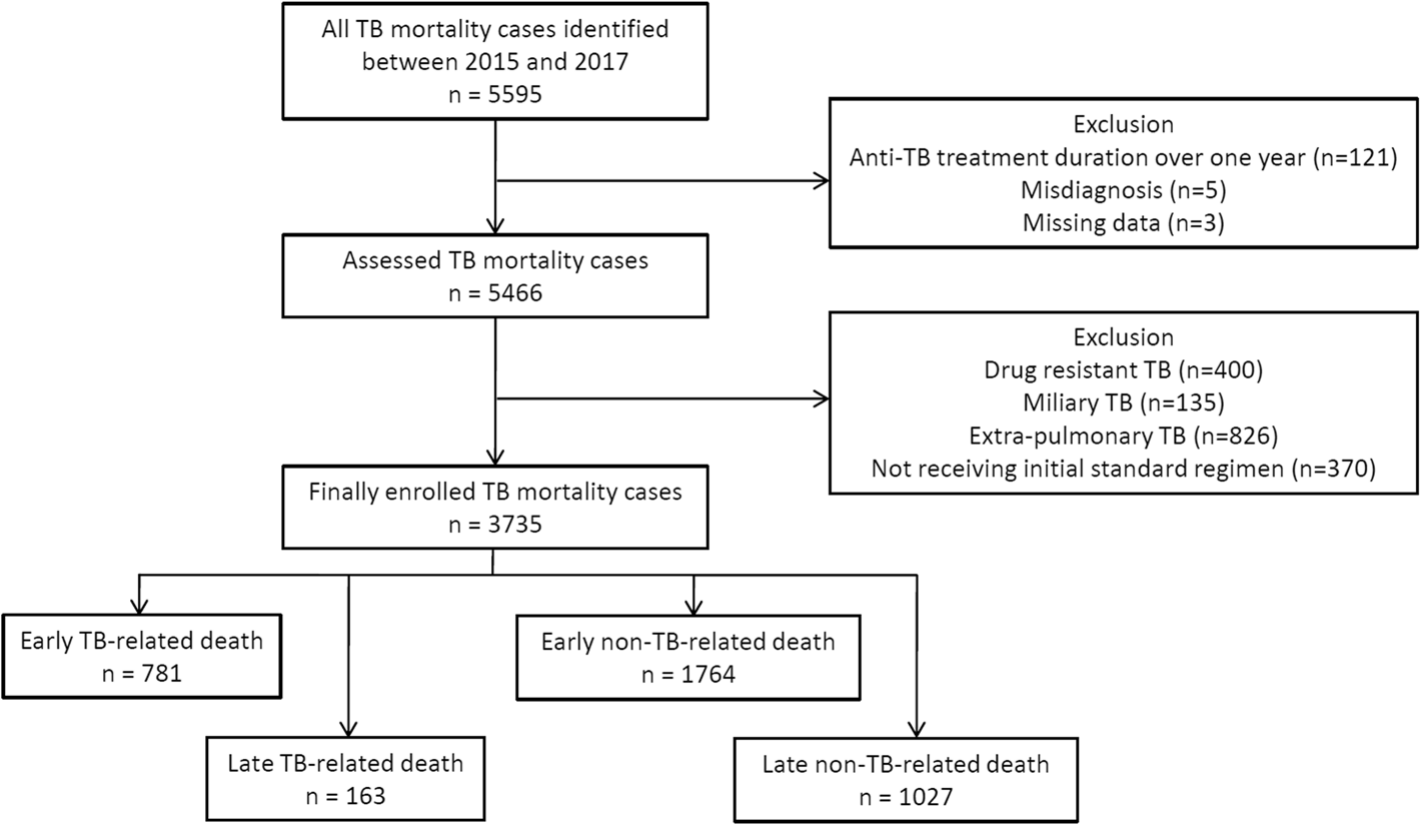
Flow chart of enrollment of tuberculosis mortality cases between 2015 and 2017, which were finally categorized based on cause (tuberculosis-related and non-tuberculosis related) and timing (early and late) TB, tuberculosis

Of 5595 TB patients with a notifiable death, we excluded patients with anti-TB treatment duration of over 1 year (*n* = 121), patients with drug-resistant TB (*n* = 400), patients with miliary TB (*n* = 135), patients with extra-pulmonary TB (*n* = 826), patients not initially receiving the standard regimen (*n* = 370), and those with misdiagnosis and missing data (n = 8). Ultimately, 3735 patients with pulmonary TB were included in this study (Fig. 1). There were 944 (25.3%) TB-related deaths and 2791 (74.7%) non-TB-related deaths. The proportion of early TB-related deaths was significantly higher than that of early non-TB-related deaths (82.7% (781/944) vs. 63.2% (1764/2791), *P* = 0.000).

The baselines characteristics of 3735 enrolled male and female patients (Tables 1 and 2). There were 2541 (68.0%) male patients, and 2935 (78.6%) mortality cases were observed in elderly patients over 65 years of age. The mean age of female patients was significantly higher than that of male patients (78.9 ± 11.7 vs. 72.1 ± 13.0, *P* = 0.000). The proportion of individuals with body mass index < 18.5 kg/m^2^ was similar between male and female patients. The proportion of prior TB history in male patients was significantly higher than that in female patients (21.5% vs. 10.9%, *P* = 0.000). Male patients were more likely to have chronic lung disease (8.0% vs. 4.7%, P = 0.000) and malignancy (24.8% vs. 12.7%, P = 0.000), and female patients had higher frequency of cardiovascular disease (8.1% vs. 13.1%, P = 0.000). The proportion of positive acid-fast bacilli (AFB) smear and culture test result were 46.9 and 69.7%, respectively.

**Table 1:** Social and demographic characteristics of enrolled TB mortality cases categorized by genders

Variables^a^	All (*n* = 3735)	Male (*n* = 2541)	Female (*n* = 1194)	*p*-value
Age group				
≤ 49 years	204 (5.5%)	157 (6.2%)	47 (3.9%)	0.000
50–59 years	365 (9.8%)	324 (12.8%)	41 (3.4%)	
60–69 years	539 (14.4%)	437 (17.2%)	102 (8.5%)	
70–79 years	1058 (28.3%)	758 (29.8%)	300 (25.1%)	
80 years ≤	1569 (42.0%)	865 (34.0%)	705 (59.0%)	
Job				
Yes	181 (4.8%)	164 (6.5%)	17 (1.4%)	0.000
No	3545 (94.9%)	2370 (93.3%)	1175 (98.4%)	
Marriage				
Yes	3244 (86.9%)	2134 (84.0%)	1110 (93.0%)	0.000
No	251 (6.7%)	218 (8.6%)	33 (2.8%)	
Education				
Primary	899 (24.1%)	508 (20.0%)	391 (32.7%)	0.008
Middle	277 (7.4%)	230 (9.1%)	47 (3.9%)	
High	385 (10.3%)	330 (13.0%)	55 (4.6%)	
University	134 (3.6%)	115 (4.5%)	19 (1.6%)	
Smoking history				
Never smoker	2413 (64.6%)	1309 (51.5%)	1104 (92.5%)	0.000
Ex-smoker	872 (23.3%)	820 (32.3%)	52 (4.4%)	
Current smoker	432 (11.6%)	397 (15.6%)	35 (2.9%)	
Alcohol history				
Non-drinker	2562 (68.6%)	1473 (58.0%)	1089 (91.2%)	0.000
Social drinker	817 (21.9%)	743 (29.2%)	74 (6.2%)	
Heavy drinker	311 (8.3%)	292 (11.5%)	19 (1.6%)	
BMI				
≥ 18.5 kg/m^2^	2130 (57.0%)	1447 (56.9%)	683 (57.2%)	0.551
< 18.5 kg/m^2^	1442 (38.6%)	989 (38.9%)	453 (37.9%)	

*BMI* body mass index, *HIV* human immunodeficiency virus

^a^ Missing data and values were not shown in this table

**Table 2:** Clinical characteristics and laboratory findings of enrolled TB mortality cases categorized by genders

Variables^a^	All (*n* = 3735)	Male (*n* = 2541)	Female (*n* = 1194)	p-value
Prior TB history				
No	3033 (81.2%)	1978 (77.8%)	1055 (88.4%)	0.000
Yes	677 (18.1%)	547 (21.5%)	130 (10.9%)	
Comorbidities				
Diabetes Mellitus	947 (25.4%)	647 (25.5%)	300 (25.1%)	0.863
Chronic lung disease	259 (6.9%)	203 (8.0%)	56 (4.7%)	0.000
Cardiovascular disease	362 (9.7%)	206 (8.1%)	156 (13.1%)	0.000
Malignancy	782 (20.9%)	630 (24.8%)	152 (12.7%)	0.000
HIV infection	12 (0.3%)	12 (0.5%)	0 (0.0%)	0.017
No reported illness	778 (20.8%)	532 (20.9%)	246 (20.6%)	0.848
Symptoms				
Cough	1641 (43.9%)	1129 (44.4%)	512 (42.9%)	0.369
Dyspnea	1250 (33.5%)	861 (33.9%)	389 (32.6%)	0.427
Haemoptysis	132 (3.5%)	94 (3.7%)	38 (3.2%)	0.424
Fever	509 (13.6%)	342 (13.5%)	167 (14.0%)	0.664
Cavitation on CXR				
Normal	110 (2.9%)	69 (2.7%)	41 (3.4%)	0.000
Non-cavitary disease	2714 (72.7%)	1781 (70.1%)	933 (78.1%)	
Cavitary disease	873 (23.4%)	666 (26.2%)	207 (17.3%)	
Lesion extension on CXR				
Normal	110 (2.9%)	69 (7.1%)	41 (3.4%)	0.624
Unilateral disease	1998 (53.5%)	1355 (53.3%)	643 (53.9%)	
Bilateral disease	1508 (40.4%)	1035 (40.7%)	473 (39.6%)	
AFB smear test				
Negative	1889 (50.6%)	1279 (50.3%)	610 (51.1%)	0.000
Positive	1753 (46.9%)	1221 (48.1%)	532 (44.6%)	
AFB culture test				
Negative	989 (26.5%)	693 (27.3%)	296 (24.8%)	0.021
Positive	2529 (67.7%)	1717 (67.6%)	812 (68.0%)	
Initial treatment regimen				
HERZ	3042 (81.4%)	2093 (82.4%)	949 (79.5%)	0.097
HRE	310 (8.3%)	203 (8.0%)	107 (9.0%)	
No treatment	383 (10.3%)	245 (9.6%)	138 (11.6%)	

*TB* tuberculosis, *CXR* chest X-ray, *AFB* acid-fast bacilli, *HREZ* combination regimen of isoniazid, rifampicin, ethambutol, and pyrazinamide, *HRE* combination regimen of isoniazid, rifampicin, and ethambutol

^a^ Missing data and values were not shown in this table

**Fig. 2:**
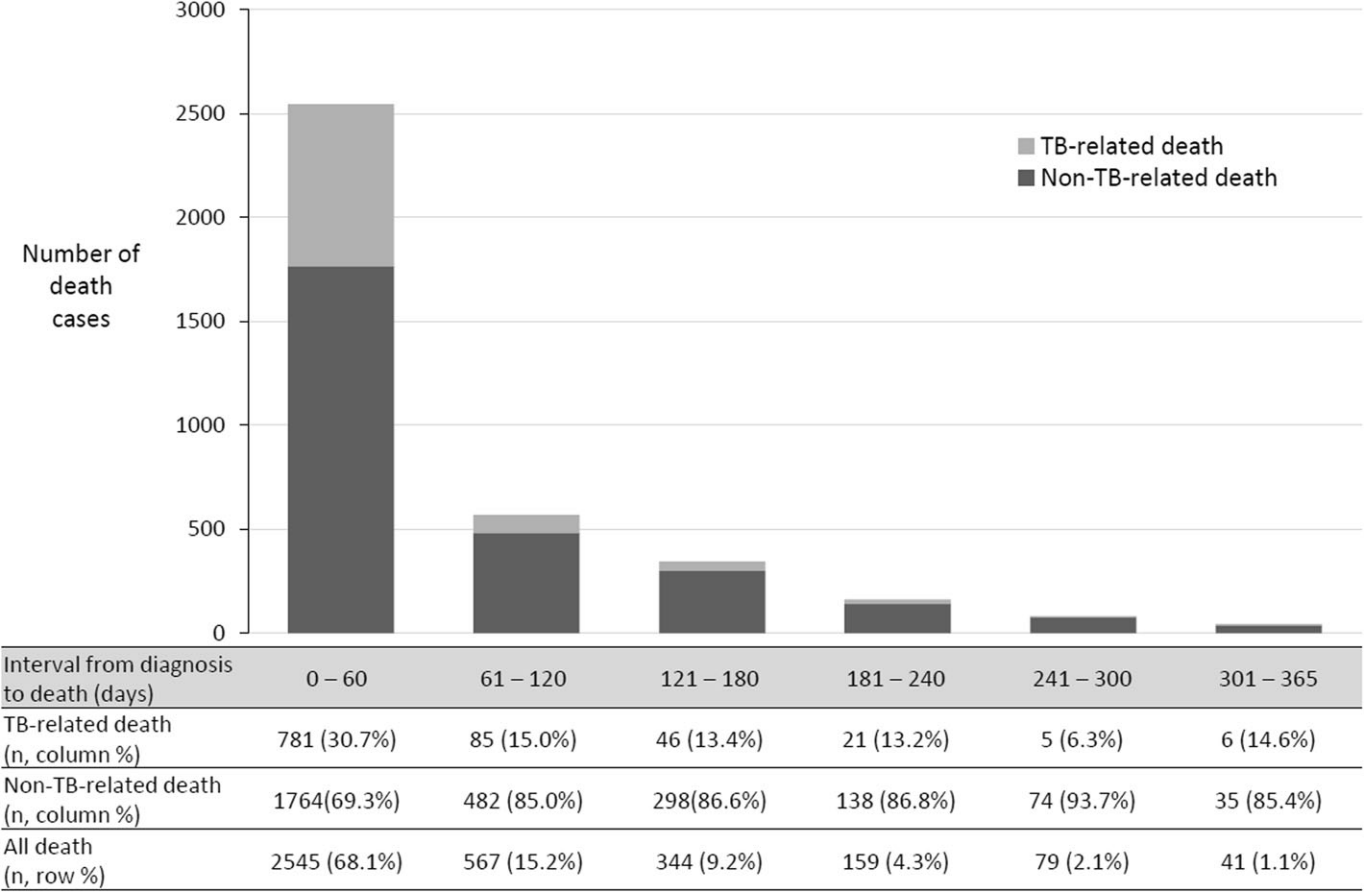
Number of tuberculosis-related, non-tuberculosis-related, and all death cases stratified by intervals between diagnosis and death. TB, tuberculosis

The cumulative number of deaths within 30 and 60 days were 1993 (53.4%) and 2545 (68.1%), respectively (Fig. 2). The median interval between diagnosis and death among all patients was 26.0 days (interquartile range 6.0–81.0 days). Among 383 (10.3%) patients who did not receive anti-TB treatment, 187 (5.0%) patients were diagnosed post-mortem. Thirty-eight (1.0%) patients died on the first day of treatment.

In multivariate analysis, current smoker (*P* = 0.023), body mass index less than 18.5 kg/m^2^ (*P* = 0.000), prior TB history (*P* = 0.002), no reported illness (*P* = 0.026), cavitary (*P* = 0.001) and bilateral disease (P = 0.000) on chest X-ray, positive AFB smear results (P = 0.000), cough (P = 0.002), and hemoptysis (*P* = 0.007) were significantly associated with TB-related death (Table 3). Cardiovascular disease, malignancy, and chest pain were associated with non-TB-related death. Calibration of this predictive model was good, as shown by a Hosmer-Lemeshow test (*P* = 0.590). Among 2791 patients who died of non-TB-related causes, acute respiratory failure (18.9%) was the most frequent cause of death, followed by pneumonia (18.7%) and malignant neoplasm (17.4%) (Table 4).

**Table 3:** Comparison of profiles of tuberculosis patients categorized by cause of death using univariate and multivariate analysis

Variables	TB-related death (*n* = 944)	Non-TB-related death (*n* = 2791)	OR (95% CI)	*p*-value	aOR^a^(95% CI)	*p*-value
Age group						
≤ 49 years	77 (8.2%)	127 (4.6%)	1		1	
50–59 years	107 (11.3%)	258 (9.2%)	0.68 (0.48–0.98)	0.040	0.81 (0.52–1.26)	0.350
60–69 years	118 (12.5%)	421 (15.1%)	0.46 (0.33–0.66)	0.000	0.86 (0.57–1.32)	0.498
70–79 years	214 (22.7%)	844 (30.2%)	0.42 (0.30–0.57)	0.000	0.76 (0.51–1.14)	0.183
80 years ≤	428 (45.3%)	1141 (40.9%)	0.62 (0.46–0.84)	0.002	1.08 (0.74–1.60)	0.686
Female	308 (32.6%)	886 (31.7%)	1.04 (0.89–1.22)	0.615	1.03 (0.83–1.28)	0.777
Current smoker^b^	161 (17.1%)	271 (9.8%)	1.89 (1.52–2.34)	0.000	1.42 (1.05–1.92)	0.023
Heavy drinker	116 (12.4%)	195 (7.1%)	0.51 (0.39–0.68)	0.000	0.94 (0.67–1.33)	0.730
BMI < 18.5 kg/m^2^	441 (50.2%)	1001 (37.2%)	1.70 (1.46–1.98)	0.000	1.38 (1.16–1.64)	0.000
Prior TB history	210 (22.4%)	467 (16.8%)	1.42 (1.19–1.71)	0.000	1.43 (1.14–1.79)	0.002
Comorbidities						
Cardiovascular disease	72 (7.7%)	290 (10.5%)	0.71 (0.55–0.93)	0.014	0.69 (0.50–0.95)	0.022
Malignancy	79 (8.4%)	703 (25.3%)	0.27 (0.21–0.35)	0.000	0.35 (0.26–0.46)	0.000
No reported illness	274 (29.2%)	504 (18.2%)	1.86 (1.57–2.21)	0.000	1.27 (1.03–1.56)	0.026
Diagnostic tests						
Cavitary disease on CXR^c^	335 (36.7%)	538 (20.1%)	2.31 (1.96–2.72)	0.001	1.43 (1.17–1.75)	0.001
Bilateral disease on CXR^d^	516 (57.1%)	992 (38.1%)	2.17 (1.86–2.53)	0.009	1.66 (1.39–1.99)	0.000
AFB smear test (+)	595 (64.7%)	1158 (42.5%)	2.48 (2.13–2.90)	0.000	1.85 (1.52–2.24)	0.000
AFB culture test (+)	683 (77.8%)	1846 (69.9%)	1.51 (1.26–1.80)	0.000	1.14 (0.91–1.43)	0.246
Symptoms						
Cough	492 (52.1%)	1149 (41.2%)	1.55 (1.34–1.80)	0.000	1.32 (1.11–1.57)	0.002
Dyspnea	349 (37.0%)	901 (32.3%)	1.23 (1.05–1.43)	0.009	1.18 (0.99–1.42)	0.072
Hemoptysis	48 (5.1%)	84 (3.0%)	1.72 (1.20–2.48)	0.003	1.79 (1.17–2.73)	0.007
Chest pain	32 (3.4%)	136 (4.9%)	0.68 (0.46–1.01)	0.058	0.67 (0.42–1.06)	0.086

TB, tuberculosis; OR, odds ratio; aOR, adjusted odds ratio; CI, confidence interval; BMI, body mass index; CXR, chest x-ray; AFB, acid-fast bacillus

% = % of cases without missing data

^a^All the variables listed in this table were used in the multivariate analysis

^b^ Never smoker was used as reference

^c^ Non-cavitary disease on chest X-ray was used as reference

^d^ Unilateral disease on chest X-ray was used as reference

**Table 4:** Modes of death among patients who died of non-tuberculosis-related cause

Categories	Non-TB-related death (*n* = 2791)
Acute respiratory failure	528 (18.9%)
Pneumonia	521 (18.7%)
Malignant neoplasm	487 (17.4%)
Septic shock	303 (10.9%)
Sudden cardiac death	242 (8.7%)
Multi-organ failure	168 (6.0%)
Acute kidney injury	60 (2.1%)
Acute hepatic failure	44 (1.6%)
Cerebrovascular disease	34 (1.2%)
Gastrointestinal bleeding	19 (0.7%)
Others internal causes	48 (1.7%)
External cause	35 (1.3%)
Senility	81 (2.9%)
Unknown	221 (7.9%)

*TB* tuberculosis

**Table 5:** Comparison of profiles of tuberculosis patients categorized by timing of death using univariate and multivariate analysis

Variables	Early death (*n* = 2545)	Late death (*n* = 1190)	OR (95% CI)	p-value	aOR^a^ (95% CI)	p-value
Age group						
≤ 49 years	144 (5.7%)	60 (5.0%)	1		1	
50–59 years	248 (9.7%)	117 (9.8%)	0.88 (0.61–1.28)	0.514	0.96 (0.64–1.44)	0.835
60–69 years	343 (13.5%)	196 (16.5%)	0.73 (0.52–1.03)	0.076	0.90 (0.61–1.32)	0.577
70–79 years	711 (27.9%)	347 (29.2%)	0.85 (0.62–1.18)	0.615	0.98 (0.69–1.41)	0.929
80 years ≤	1099 (43.2%)	470 (39.5%)	0.97 (0.71–1.34)	0.708	1.01 (0.71–1.44)	0.968
Female	827 (32.5%)	367 (30.8%)	1.08 (0.93–1.25)	0.312	0.90 (0.75–1.08)	0.238
Current smoker^b^	723 (61.6%)	1690 (66.7%)	0.72 (0.62–0.85)	0.000	0.76 (0.59–0.99)	0.039
Body mass index < 18.5 kg/m^2^	1030 (42.6%)	412 (35.7%)	1.34 (1.16–1.55)	0.000	1.26 (1.08–1.48)	0.003
Comorbidities						
Diabetes mellitus	321 (27.1%)	626 (24.8%)	0.89 (0.76–1.04)	0.128	1.01 (0.85–1.21)	0.886
Malignancy	1030 (18.1%)	412 (27.4%)	0.58 (0.50–0.69)	0.000	0.71 (0.59–0.89)	0.000
No reported illness	580 (23.0%)	198 (16.7%)	1.48 (1.24–1.77)	0.000	1.36 (1.10–1.68)	0.005
Diagnostic tests						
Bilateral disease on CXR^c^	414 (36.7%)	1094 (46.0%)	1.47 (1.27–1.70)	0.000	1.30 (1.11–1.52)	0.001
AFB smear test (+)	1254 (50.6%)	499 (42.9%)	1.36 (1.18–1.56)	0.000	1.30 (1.11–1.52)	0.001
Symptoms						
Dyspnea	924 (36.3%)	326 (27.4%)	1.51 (1.30–1.76)	0.000	1.52 (1.29–1.79)	0.000
Fever	369 (14.5%)	140 (11.8%)	1.27 (1.03–1.57)	0.023	1.21 (0.96–1.55)	0.104

OR, odds ratio; aOR, adjusted odds ratio; CI, confidence interval; CXR, chest X-ray; AFB, acid-fast bacillus

% = % of cases without missing data

^a^All the variables listed in this table were used in the multivariate analysis

^b^ Never smoker was used as reference

^c^ Unilateral disease on chest X-ray was used as reference

In comparison with individuals with late death, individuals with early death had significantly higher proportion of body mass index < 18.5 kg/m^2^ (*P* = 0.003), no reported illness (*P* = 0.005), bilateral disease on chest X-ray (*P* = 0.001), positive AFB smear result (P = 0.001), and dyspnea (*P* = 0.000) (Table 5). Current smoker status (*P* = 0.039) and malignancy (P = 0.000) were associated with late death. Model adequacy was checked using a Hosmer-Lemeshow test (*P* = 0.057).

**Fig. 3:**
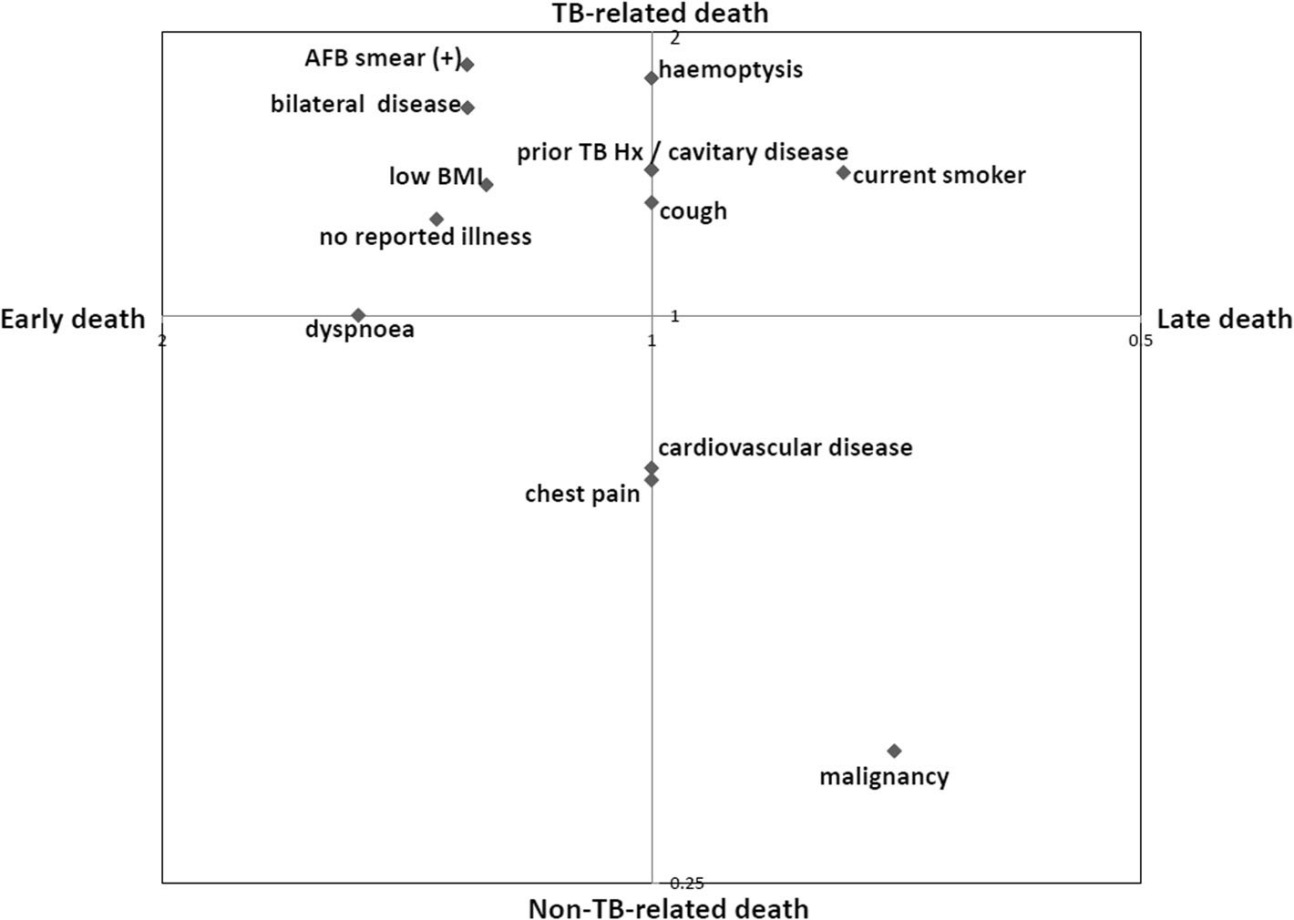
Log-Log plot describing variables, which were significantly associated with subsets of TB death (TB-related, non-TB-related, early and late deaths). TB, tuberculosis; AFB, acid-fast bacillus; BMI, body mass index; Hx, history

After identifying variables significantly associated with each predefined subsets of TB mortality, we plotted a log-log graph describing factors related to early TB-related and late non-TB-related deaths (Fig. 3). The factors in the right upper quadrant, such as low body mass index, no reported illness, bilateral disease on chest X-ray, and positive AFB smear result, were associated with both TB-related and early death. In contrast, malignancy was associated with both non-TB-related death and late death.

## Discussion

This large cross-sectional study assessed 3735 TB mortality cases that occurred during anti-TB treatment among adult patients with pulmonary TB in South Korea. According to our study, three of every four deaths occurring during anti-TB treatment were attributed to non-TB-related causes such as acute respiratory failure and malignancy. We also observed a high number of TB mortality cases among elderly patients in this study. South Korea has become an aged society, and the epidemiologic pattern within the past few decades has changed from infectious to chronic non-communicable diseases that cause individuals (particularly elderly individuals) to be at a greater risk of developing TB [5]. More attentions to this elderly population are needed, and accordingly, South Korea is currently preparing a comprehensive management plan for its elderly population, which is a key vulnerable group [3].

There are several factors that contribute to the enormous TB death toll worldwide [17]. Although global initiatives address the importance of malnutrition [18] and smoking [19], which have been clearly linked with excess TB mortality, such key determinants of TB mortality are still under-emphasized at the country level. A weak and underinvested public health system, especially in low-income countries, is another important issue, and has led to a suboptimal cascade of care for TB patients [20]. Since 2011, South Korea has increased budgets and strengthened patient management policies together with a PPM collaboration model, which led to significant decreases in TB incidence [3]. However, a high and stagnant TB mortality rate poses a great challenge to the national TB control program in South Korea. Our study findings may allow for planning of effective interventions to strengthen patient-centered care and improve patient survival.

As illustrated in the results, low body mass index, no reported illness, bilateral disease on chest X-ray, and positive AFB smear result were significantly associated with early and TB-related death. Because we aimed to identify mortality cases that could be controlled and prevented through appropriate management, we excluded TB cases presumed to be hard to treat, such as cases involving immunocompromised host, drug resistance, and disseminated disease. Cases of spinal or miliary disease reflect compromised immunity and are difficult to manage even with proper intervention. Patients with drug resistance are prone to poor medication adherence and severe adverse reactions related to non-TB-related death [21]. Based on our results, we can propose that early interventions should target patients with severe TB disease and malnutrition, in order to improve TB mortality.

Proper diagnosis and rapid treatment of patients with infectious TB remains the cornerstone of a TB control program [22]; however, 10% of patients enrolled in our study died without receiving anti-TB drugs, and 5% of patients were diagnosed post-mortem. TB control is also complicated by post-mortem diagnosis, as these patients likely had multiple encounters with healthcare facilities while infected with unrecognized TB [23]. Automated nucleic acid amplification tests such as Xpert MTB/RIF assay are increasingly deployed in many countries as the point-of-care diagnostic test for TB [24]. Recently, South Korea has expanded the coverage of national health insurance for the Xpert MTB/RIF assay. Elderly patients who cannot produce adequate sputum specimen would benefit from the implementation of such a sensitive tool, which could reduce missed or delayed diagnosis.

Due to the difficulties inherent to differentiating TB-related death from non-TB-related death, only a small number of published studies have examined risk factors for TB-related death. A Taiwanese study [25] found that extrapulmonary, miliary, and pneumonic radiographic patterns were independent risk factors for TB-related death when compared to survivors. A single-center study from South Korea [11] suggested that early TB-related deaths were mainly attributable to delayed diagnosis. Another recent study that evaluated TB mortality revealed that an underlying chronic condition, lower hemoglobin level, and acute respiratory failure were independent risk factors for TB-related death among non-elderly patients with miliary TB [12]. In order to meet the target goal of reducing TB mortality in South Korea, clinicians should be educated on the identification of preventable risk factors for TB-related death. In addition, South Korea estimates for TB mortality are based on National Statistical Office data. These data reflect only TB-related death, because the cause of death on the death certificate is used to identify the mortality case in accordance with the WHO recommendations [26]. Accuracy and utility of cause of death data from death certificates are uncertain and often questionable. The current study is valuable in that it is the first attempt to assess TB mortality using a large nationwide database in South Korea. An accurate estimate of mortality enables a clearer understanding of the burden and cost of TB and can inform national TB control strategies to improve mortality [27].

Our study has several limitations. First, we only described and compared characteristics of predefined subsets of TB mortality cases, and data of TB survivors were unavailable for the statistical analysis. The WHO states that for country-specific purposes, deaths due to TB and deaths due to other causes can be separated in the treatment outcomes section [16]. In South Korea, both TB-related and non-TB-related deaths are reported in the KNTSS. Although our results cannot be extrapolated to the general TB population, they remain valuable for the purpose of assessing the national TB status and planning necessary interventions. Second, those who visited PPM hospitals before death were included for this analysis, which also limits the generalizability. More information regarding the characteristics and treatment outcomes of TB patients managed in non-PPM hospitals are essential to reducing overall TB mortality. Third, we extracted data regarding the causes of death from case report forms completed by TB nurses at PPM hospitals, who identify the causes by reviewing and comparing two available sources (medical charts and death certificates). However, in routine clinical practice, it is hard to identify the correct mode of death, especially for elderly individuals. We could not implement a robust verification method to validate the information regarding the mode of death, because of the large cross-sectional design of the study. This may have caused a potential misclassification bias. In addition, cases of out-of-hospital mortality are collected by interviewing the guardian. The mode of death was unknown or forgotten in 10% of cases and resulted in the underestimation of TB-related death. Fourth, because clinical data for this analysis were collected from a database designed for TB surveillance, we could not obtain all clinical data related to death, such as laboratory findings and vital signs. A detailed history of TB diagnosis and treatment were not captured. For example, non-adherence to anti-TB drugs and delays to diagnosis and treatment were known to be associated with TB death [28]. Because adverse reactions to anti-TB drugs are distinct concerns for elderly patients with TB [29], further analysis investigating adverse drug reactions and mortality could be helpful to elucidate the high proportion of mortality among the elderly population. Lastly, because of the cross-sectional study design and unavailability of data regarding TB survivors, we could not measure outcomes over time or apply time-to-event analysis, which might have provided a more complex understanding of TB mortality.

## Conclusions

A high proportion of TB death was observed among elderly patients, indicating that special attention to this population is necessary. Our results revealed that most of the deaths during anti-TB treatment were attributed to non-TB related causes, such as acute respiratory failure, malignancy, and cardiovascular diseases. Although a timely diagnosis may not prevent most non-TB related deaths, early treatment could reduce transmission risk, which is valuable in the public health perspective. Many TB-related deaths occurred during the intensive phase, particularly within the first month. Further studies identifying risk factors for different causes of TB death at different phases of anti-TB treatment are warranted for early identification of risk factors and targeted intervention, which may reduce early TB-related deaths. Any such intervention requires the collaboration of public and private sectors to maximize its effect.

## Data Availability

The ownership of the primary datasets lies with the Korea Centers for Disease Control and Prevention (KCDC). The datasets used and/or analyzed during the current study are available on reasonable request after obtaining permission from the KCDC in advance. Joosun Lee of the KCDC should be contacted for the request accessing the raw data.

## Abbreviations

AFB: Acid-fast bacilli
PPM: Private-public mix
SPSS: Statistical package for the social sciences
TB: Tuberculosis
WHO: World Health Organization
